# Effect of exacerbations on health status in subjects with chronic obstructive pulmonary disease

**DOI:** 10.1186/1477-7525-7-69

**Published:** 2009-07-22

**Authors:** Koichi Nishimura, Susumu Sato, Mitsuhiro Tsukino, Takashi Hajiro, Akihiko Ikeda, Hiroshi Koyama, Toru Oga

**Affiliations:** 1 Department of Respiratory Medicine, Murakami Memorial Hospital, Asahi University, Gifu, Japan; 2 Department of Respiratory Medicine, Graduate School of Medicine, Kyoto University, Kyoto, Japan; 3 Department of Respiratory Medicine, Hikone Municipal Hospital, Hikone, Japan; 4 Department of Respiratory Medicine, Tenri Hospital, Tenri, Japan; 5 Department of Respiratory Medicine, Nishi-Kobe Medical Center, Kobe, Japan; 6 General Internal Medicine, National Hospital Organization Kyoto Medical Center, Kyoto, Japan; 7 Department of Respiratory Care and Sleep Control Medicine, Graduate School of Medicine, Kyoto University, Kyoto, Japan

## Abstract

**Background:**

Acute exacerbations may cause deteriorations in the health status of subjects with chronic obstructive pulmonary disease (COPD). The present study prospectively evaluated the effects of such exacerbations on the health status and pulmonary function of subjects with COPD over a 6-month period, and examined whether those subjects showed a steeper decline in their health status versus those subjects without exacerbations.

**Methods:**

A total of 156 subjects with COPD (mean age 71.4 ± 6.3 years) were included in the analysis. At baseline and after 6 months, their pulmonary function and health status were evaluated using the Chronic Respiratory Disease Questionnaire (CRQ) and the St. George's Respiratory Questionnaire (SGRQ). An acute exacerbation was defined as a worsening of respiratory symptoms requiring the administration of systemic corticosteroids or antibiotics, or both.

**Results:**

Forty-eight subjects experienced one or more exacerbations during the 6-month study period, and showed a statistically and clinically significant decline in Symptom scores on the SGRQ, whereas subjects without exacerbations did not show a clinically significant decline. Logistic multiple regression analyses confirmed that the exacerbations significantly influenced the Fatigue and Mastery domains of the CRQ, and the Symptoms in the SGRQ. Twelve subjects with frequent exacerbations demonstrated a more apparent decline in health status.

**Conclusion:**

Although pulmonary function did not significantly decline during the 6-month period, acute exacerbations were responsible for a decline in health status. To minimize deteriorations in health status, one must prevent recurrent acute exacerbations and reduce the exacerbation frequencies in COPD subjects.

## Background

Acute exacerbations are associated with considerable symptomatic and physiological deterioration, and can worsen the health status in subjects with chronic obstructive pulmonary disease (COPD) [1,2]. Seemungal *et al*. [3] reported that subjects with frequent COPD exacerbations during the preceding year had significantly higher St. George's Respiratory Questionnaire (SGRQ) [4] scores than those with less frequent exacerbations. Assessments during acute exacerbations demonstrated that the health status worsened relative to subjects with stable COPD [4-12], and that the recovery period was longer, even in subjects without further exacerbations, since a single exacerbation has a sustained effect on health status [7]. Inhaled corticosteroids (ICS) reduced the frequency of acute exacerbations and also reduced the rate of decline in the health status, since frequent exacerbations were associated with a more rapid rate of deterioration in health status [13-15]. Furthermore, several studies [8-12,16,17] showed that exacerbations were associated with a significant worsening in the health status of subjects with COPD as measured by SGRQ scores.

Since acute COPD exacerbations could accelerate the decline in health status in stable COPD subjects, and these subjects with exacerbations would show a steeper decline in health status versus subjects without exacerbations. Therefore, the present study prospectively evaluated the effects of exacerbations on the health status and pulmonary function of subjects with COPD over a 6-month period. The health status was measured by two widely-used disease-specific questionnaires, the Chronic Respiratory Disease Questionnaire (CRQ) [18] and the SGRQ[4].

## Methods

### Subjects

A total of 156 consecutive subjects with COPD (149 males) were enrolled in this study from the Kyoto University Hospital outpatient clinic from October 1999 to March 2000. The entry criteria for this study were as follows: (1) a clinical diagnosis of stable COPD as defined by the American Thoracic Society [19]; (2) a maximal forced expiratory volume in one second (FEV_1_) <80% of the predicted value for all measurements made during the previous 6 months; (3) a maximal FEV_1_/forced vital capacity (FVC) ratio <0.7; (4) a smoking history >20 pack-years; (5) prior, regular treatment at our clinic for more than 6 months; (6) no exacerbations of airflow limitation over the preceding 6 weeks; (7) no history suggestive of asthma; and (8) no severe comorbidities.

An acute exacerbation was defined as a worsening of respiratory symptoms that required treatment with oral corticosteroids or antibiotics, or both [13,14]. Pulmonary function tests, blood gas analyses, and health status were measured on the same day at baseline, and again after 6 months when the subjects were stable. If an exacerbation occurred on the second evaluation day after 6 months, then the subjects were rescheduled for another evaluation at 6 weeks after the development of respiratory infections or exacerbations. All measurements were performed after a 6-week exacerbation-free period. All subjects attended the second visit, and were considered to be valid.

### Pulmonary function tests

Inhaled bronchodilators were withheld for at least 12 h before the pulmonary function tests. All spirometric flow-volume curves for determining the FEV_1 _and FVC were recorded according to the methods described in the American Thoracic Society 1994 update [20], using a spirometer (AUTOSPIRO AS-600, Minato Medical Science Co. Ltd., Osaka, Japan) which was calibrated with a 3.0 L syringe. The FEV_1 _and FVC were assessed before, and at 15 and 60 minutes after the inhalation of bronchodilators (80 μg of ipratropium bromide plus 400 μg of salbutamol), using a metered-dose inhaler with a spacer device (InspirEase^®^; Schering-Plough K. K., Osaka, Japan). Except for the FEV_1 _and FVC, all pulmonary function tests were performed with a Chestac-55V (Chest MI Corp., Tokyo, Japan) before bronchodilation. The residual volumes (RV) and total lung capacity (TLC) were measured by a helium dilution method. The carbon monoxide transfer coefficient (KCO) was measured by the single-breath method. The predicted values for the pulmonary function measurements were calculated according to the Japan Society of Chest Diseases' proposal [21]. The partial pressures of arterial oxygen (PaO_2_) and carbon dioxide (PaCO_2_) were measured at rest using a blood gas analyzer (ABL 620, Radiometer Medical A/S, Copenhagen, Denmark).

### Health status assessments

The health status of each subject was assessed by two major, previously-validated Japanese versions of [22], disease-specific questionnaires: the CRQ [18] and SGRQ [4]. The two questionnaires were self-administered at baseline, and again after 6 months under the supervision of the investigators, in the same order, in booklet form. An investigator (T.O.) reviewed the surveys to ensure that the subjects did not unintentionally omit questions.

The CRQ consists of 20 items and four domains (Dyspnea, Fatigue, Emotional function, and Mastery), and each question was presented as a seven-point scale [18]. Each domain of the CRQ was scored as the sum of these points, and higher scores represent a better health status. The total score, as represented by the sum of the scores from these four domains, was also calculated. A change in score of 0.5 points per question is consistent with a clinically significant change in the subject [23].

The SGRQ consists of 50 items and three components (Symptoms, Activity, and Impacts) [4]. The three components of the SGRQ were transformed into a score from 0 to 100. Higher scores indicate a poorer health status. A change in the score of 4 units is consistent with a clinically significant change in the subject [24].

### Statistical analyses

Statistical analyses were performed using SPSS 6.1 software (SPSS Institute, Chicago, IL). The results are presented as means ± SD, unless otherwise stated. Comparisons of continuous variables between subjects with exacerbations versus without exacerbations were performed using *t *tests, and discrete variables were compared using Chi-square tests. Data that were not normally distributed were subjected to a Mann-Whitney U test. Comparisons of changes in physiological parameters were analyzed by a paired *t *test. Wilcoxon sign-rank tests were used to compare changes in the health status. We performed stepwise multiple regression analyses to predict the changes in each health status score. Furthermore, we also performed stepwise multiple logistic regression analyses to identify the influence of risk factors (including acute exacerbations) on the health status deterioration, which was defined as exceeding the minimal significant differences (0.5 units per question for the CRQ and 4 units for the SGRQ). In both regression analyses, the independent variables included the frequency of acute exacerbation, age, pulmonary function (FEV_1_, KCO, and RV/TLC) at baseline, smoking status (current smoking = "1", and former smoking = "0"), and each baseline score of the relevant domain. The significance level of all analyses was set at 5%.

## Results

The patient characteristics and health status at baseline are presented in Table 1. The mean age of all subjects was 71.4 years, with a mean pre-bronchodilator FEV_1 _of 0.97 L (38.1% predicted). Twenty-eight subjects were current smokers at baseline. One hundred fifty-four (98.7%) subjects regularly received an inhaled bronchodilator (anti-cholinergic agent and beta-2 agonist), and seventy-nine (50.6%) subjects received additional high dose ICS (beclomethasone dipropionate) at 1600 μg daily, and nine (5.8%) subjects also received oral corticosteroids at baseline. Two subjects were managed with long-term domiciliary oxygen therapy. All subjects visited our outpatient clinic for regular examinations.

**Table 1:** Characteristics and health status of COPD subjects at baseline according to the exacerbation status during the 6-month follow-up*

	With exacerbation (n = 48)	Without exacerbation (n = 108)	P value
Gender (M/F)	46/2	103/5	0.90
Age, yrs	71.4 ± 7.0	71.4 ± 6.0	0.95
pre-bronchodilator FEV_1_, L	0.83 ± 0.22	1.04 ± 0.37	< 0.001
pre-bronchodilator FEV_1_, %pred.	32.8 ± 9.1	40.5 ± 13.3	<0.001
pre-bronchodilator FVC, L	2.06 ± 0.42	2.26 ± 0.66	0.049
post-bronchodilator FEV_1_, L	1.04 ± 0.26	1.26 ± 0.43	<0.001
post-bronchodilator FEV_1_, %pred.	40.7 ± 10.9	49.3 ± 15.4	<0.001
post-bronchodilator FVC, L	2.50 ± 0.46	2.67 ± 0.66	0.10
Current/former-smokers	10/38	18/90	0.53
Using inhaled corticosteroids, %	54.2	49.1	0.57
TLC, L	5.78 ± 0.88	5.60 ± 1.00	0.28
RV/TLC, %	50.4 ± 7.3	47.0 ± 8.7	0.02
K_CO_, mmol·min^-1^·kPa^-1^·L^-1^	0.83 ± 0.38	0.99 ± 0.39	0.02
PaO_2_, kPa	9.71 ± 1.20	9.81 ± 1.17	0.62
PaCO_2_, kPa	5.48 ± 0.41	5.40 ± 0.52	0.37
CRQ**			
Dyspnea (5–35)	25.4 ± 5.4	27.0 ± 5.2	0.09
Fatigue (4–28)	19.0 ± 5.3	20.3 ± 5.4	0.19
Emotion (7–49)	37.4 ± 8.5	39.4 ± 8.6	0.18
Mastery (4–28)	22.0 ± 4.4	22.4 ± 4.6	0.60
Total (20–140)	103.8 ± 20.2	109.0 ± 20.3	0.14
SGRQ (0–100)^†^			
Symptoms	53.7 ± 20.7	45.2 ± 22.8	0.03
Activity	54.4 ± 19.8	43.1 ± 23.3	0.003
Impacts	32.8 ± 18.9	26.9 ± 19.1	0.08
Total score	44.5 ± 17.7	36.2 ± 19.4	0.013

* The data are presented as mean ± SD unless otherwise stated. ** Higher scores indicate a better quality of life on the CRQ. † Higher scores indicate a poorer health status on the SGRQ.

Forty-eight (30.8%) subjects had one or more exacerbations during the 6-month study period. Twelve of these subjects (25.0%) had two or more exacerbations. A total of 64 exacerbations were identified. The mean frequency of exacerbations was estimated at 0.82 per subject per year.

At baseline, those subjects with exacerbations showed a significantly higher RV/TLC and lower pre-bronchodilator FEV_1_, post-bronchodilator FEV_1_, pre-bronchodilator FVC, and KCO values than those subjects without exacerbations (Table 1). Subjects with exacerbations also showed a significantly worse health status in the Symptoms, Activity and total scores on the SGRQ than subjects without exacerbations (Table 1). Seventy-nine subjects were on ICS, and acute exacerbations occurred in 26 of these subjects (32.9%). On the other hand, 77 subjects were not taking ICS, and exacerbations occurred in 22 of these subjects (28.5%). Logistic regression analysis revealed that ICS did not influence the exacerbations (odds ratio = 1.07, 95%CI; 0.71–1.61). From an analysis of the baseline data obtained from the SGRQ, on the second question regarding phlegm, twenty-five subjects had chronic phlegm on "several" or "most days" per week, and eight of them (32%) had exacerbations. This rate of exacerbation was not significantly different from subjects without symptoms suggestive of chronic bronchitis. There were no significant differences between subjects with and without exacerbations in terms of age, arterial blood gas tension, or the proportion of subjects currently smoking.

**Figure 1:**
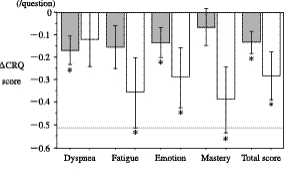
**Changes in the CRQ scores over 6 months in subjects with (open bars) and without acute exacerbations (gray bars)**. Mean scores ± SE in comparison to the baseline are presented. The broken line indicates a clinically significant deterioration in health status. A lower score indicates a deterioration in health status *: p < 0.05 versus baseline.

**Figure 2:**
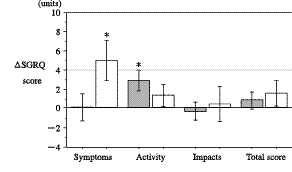
**Changes in the SGRQ scores over 6 months in subjects with (open bars) and without acute exacerbations (gray bars)**. Mean scores ± SE in comparison to the baseline are presented. The broken line indicates a clinically significant deterioration in health status. A higher score indicates a deterioration in health status.*: p < 0.05 versus baseline.

Figures 1 and 2 showed the changes in the health status of each group of subjects. In subjects without exacerbations, statistically significant (p < 0.05) declines in health status were observed in two domains (Dyspnea and Emotion), the total scores of the CRQ and in the Activity scores of the SGRQ. In subjects with exacerbations, statistically significant declines in health status were observed in three domains (Fatigue, Emotion, and Mastery), the total scores of the CRQ and the Symptoms scores of the SGRQ. Subjects with exacerbations showed a clinically significant decline (>4 units) during the 6-month study period in their SGRQ Symptom scores. In those subjects with exacerbations, the spirometric values and arterial blood gas tension did not change significantly over the 6-month study period. In those without exacerbations, only small but significant declines in spirometric values were observed (post-bronchodilator FEV_1 _and pre-bronchodilator FVC between-subject comparisons, both p values = 0.04). However, these differences were not statistically significant after adjusting for physical constitution.

The results from the stepwise multiple regression analyses used to predict the changes in health status scores are presented as determinations of coefficients (R^2^) (Additional file 1, Table S1). The baseline scores from each subscale significantly predicted the subsequent changes in each score, except for the total score of the CRQ. Following the baseline scores, the frequency of exacerbations significantly accounted for the changes in the Mastery of the CRQ (R^2 ^= 0.03) and the Symptoms of the SGRQ (R^2 ^= 0.05), and the RV/TLC accounted for the changes in the Impacts of the SGRQ (R^2 ^= 0.03).

The results from logistic regression analyses are presented (Additional file 1, Table S2) as adjusted odds ratios, which may be interpreted as measurements of independent relative risk factors. The number of subjects whose change score exceeded the MCID was 43 (Dyspnea), 59 (Fatigue), 48 (Emotion), 58 (Mastery) and 39 (Total) on the CRQ, and 68 (Symptoms), 71 (Activity), 46 (Impacts) and 51 (Total) on the SGRQ. An increase in the occurrence of an acute exacerbation, in other words additional exacerbations, caused a significant deterioration in the health status in the Fatigue (odds ratio (OR) = 1.77, p = 0.02) and Mastery (OR = 1.92, p = 0.01) domains of the CRQ, and in the Symptom scores (OR = 2.11, p = 0.01) in the SGRQ. Only the SGRQ Symptom scores with worse baseline scores showed a significant influence on the decline in health status (OR = 0.97, p < 0.001). Thus, the above two types of regression analyses (Additional file 1, Tables S1 and S2) confirmed that the frequency of exacerbations significantly affected the decline in health status.

**Figure 3:**
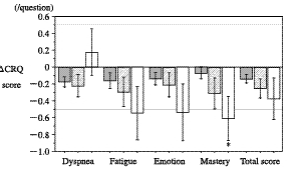
**Changes in the CRQ scores over 6 months in subjects without acute exacerbations (gray bars), subjects with one exacerbation (hatched bars), and those with two or more exacerbations (open bars)**. The results are presented as means ± SE. *: p < 0.05 versus baseline.

**Figure 4:**
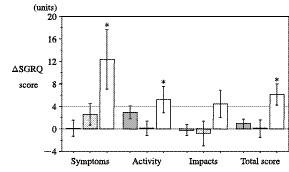
**Changes in the SGRQ scores over 6 months in subjects without acute exacerbations (gray bars), subjects with one exacerbation (hatched bars), and those with two or more exacerbations (open bars)**. The results are presented as means ± SE. *: p < 0.05 versus baseline.

Additional analyses were performed on those subjects with frequent exacerbations. Subjects with two or more exacerbations (n = 12) showed clinically significant declines in health status in the Fatigue, Emotion and Mastery domains of the CRQ (-0.54, -0.54, and -0.60/question, respectively) (Figure 3), and in all three components and the total score of the SGRQ (Symptoms: 12.4, Activity: 5.1, Impacts: 4.4, and total SGRQ: 6.1 units, respectively) (Figure 4). Spirometric examinations showed a decline in the pre-bronchodilator and post-bronchodilator FEV_1 _(0.05 ± 0.14 and 0.05 ± 0.12 L, respectively), but these changes were not statistically significant. There were no significant differences in the frequency of acute exacerbations between those subjects who received ICS and those who did not.

## Discussion

To our knowledge, this is the first study in the literature that prospectively examines and clearly demonstrates the influence of acute exacerbations on the health status deterioration in subjects with COPD. We examined 156 COPD subjects, and found that 48 experienced exacerbations during the 6-month study period. Although changes in pulmonary function were not observed, the exacerbation effects on the health status were determined to be statistically significant. Moreover, frequent exacerbations were associated with clinically significant disturbances in health status.

Comparisons between the baseline values and values after 6 months revealed that subjects with exacerbations showed a 5-unit decline in the Symptom scores in the SGRQ. In contrast, subjects without exacerbations did not show any changes in excess of the minimal clinical significant decline (0.5 units per question for the CRQ and 4 units for the SGRQ). Multiple regression analyses confirmed that the exacerbations significantly predicted the changes in the Mastery of the CRQ and Symptoms of the SGRQ, independent of their baseline scores, and logistic regression analyses confirmed a statistically significant effect of the exacerbations on the health status deterioration in the Fatigue and Mastery of the CRQ and the Symptoms of the SGRQ. These results support our hypothesis that subjects experiencing exacerbations would show a steeper decline in health status than those without exacerbations. Despite no significant changes in the physiological indices, including the FEV_1_, an increased frequency of acute exacerbations was associated with a deterioration in health status during the 6-month study period.

We also found that subjects who had two or more exacerbations showed statistically and clinically significant declines in three domains of the CRQ (Fatigue, Emotion, and Mastery), and in all three components and the total scores of the SGRQ (Figure 3 &4). These results are consistent with previous reports [3,15-17], which indicated that patients with frequent exacerbations showed a poorer health status. Spencer *et al*, [7] reported that a single infective exacerbation of chronic bronchitis has a large and sustained effect on the health status, and although the initial recovery is fast, the convalescence period is long even in subjects who did not experience further exacerbations. If recurrent exacerbations occur over a short period, then the deterioration in health status may be enhanced. Delayed recoveries in the health status may be cumulative, and therefore only subjects with frequent exacerbations may demonstrate a clinically significant decline in health status in the present study. These results are compatible with those from previous studies [15-17], including the ISOLDE study [13,14]. Exacerbations may have detrimental and cumulative effects on health status, and thus the frequency of exacerbations is a significant factor that leads to a poor health status. Therefore, to prevent deteriorations in the health status, it is important to prevent recurring acute exacerbations.

The Lung Health Study [25] reported that lower respiratory illnesses would promote a decline in the FEV_1 _in smokers with COPD, whereas a similar effect was not observed in sustained quitters. Donaldson *et al*, [26] reported that subjects who suffered frequent exacerbations experienced a significantly greater decline in their FEV_1 _than subjects who had infrequent exacerbations. However, in the present study, the exacerbations did not significantly influence any physiological parameters over the 6-month period, although exacerbations had a significant effect on the long-term deterioration in health status. Even in patients with frequent exacerbations, the FEV_1 _did not show any statistically significant changes over the 6-month study period. Although the negative results in the FEV_1 _decline in the present study are likely due to the short study period and the small number of enrolled subjects, the results of the present study suggest that acute COPD exacerbations have a more potent influence on the long-term deterioration in health status than on pulmonary function. Although the FEV_1 _only weakly correlates to health status [22], the exacerbation effects on health status versus physiological parameters clearly differ.

This study has several limitations. Since we intended to assess the effects of exacerbations, we established a study interval of 6 months. Anthonisen *et al*. [27] reported that the incidence of exacerbations was 1.1 per subject per year, so we expected that almost one-half of the subjects would have experienced an acute exacerbation during the 6-month study period. However, acute exacerbations occurred in only 31% of the subjects. If we had established a more prolonged study interval, then more subjects may have experienced exacerbations. Second, concerning the definition of an exacerbation, we selected the definition of an acute exacerbation of COPD similar to that utilized in the ISOLDE trial [14,28]. However, definitions of an acute exacerbation in the literature vary [2,19,28-30], indicating that there is no clear consensus on the definition of an exacerbation. The application of a different definition of exacerbation may explain the lower exacerbation rate in our study as compared to a previous report by Seemungal *et al*. [3,26]. According to an article by Burge and Wedzicha [28], the use of daily cards is recommended to prospectively ascertain acute exacerbations. Third, there were significant differences in baseline status between subjects with and without exacerbations. Notably, differences in the baseline health status reached minimal clinical significance, and may have influenced the changes in health status. However, except for the baseline score of the Symptoms in the SGRQ, the logistic multiple regression analyses did not show any significant effect on this factor, even though it was significantly different at baseline between subjects with and without exacerbations. In this analysis, we could deny the influence of differences between each group on the physiological factors at baseline. Indeed, the differences in the FEV_1 _at baseline may reflect higher risk factors for exacerbations in both the health status and the physiological status, and these results are compatible with the close relationship between the severity of COPD and the high risk of exacerbations shown in previous studies [31,32]. However, in the present study, we focused on the effect of exacerbations on the decline in health status, and therefore an analysis to identify the risk factors for acute exacerbations was not performed. Although only one-third of the exacerbations have been reported, and both reported and unreported exacerbations have an impact on health status [11,12]. The last limitation to our study is that unreported exacerbations were not included [11,12,33].

## Conclusion

In conclusion, we demonstrated that acute exacerbations require the use of additional medication, even in an outpatient clinic, that could be detected statistically over a 6-month period. Although the FEV_1 _did not significantly decline, acute COPD exacerbations were responsible for the decline in health status. In addition, when acute exacerbations occur frequently, the health status deterioration may be enhanced and may exceed the minimal clinically significant decline in the CRQ and SGRQ scores during the 6-month study period. It is therefore necessary to prevent recurrent exacerbations in order to minimize deteriorations in the health status. Moreover, since the unfavourable aspects caused by exacerbations may be related to health status deterioration, health-care professionals should pay special attention to the health status scores in COPD subjects, and especially scores following exacerbations.

## Competing interests

The authors declare that they have no competing interests.

## Authors' contributions

KN was a physician responsible for all the participants, set out the study design, and prepared the final manuscript. SS collected the data and prepared the initial manuscript. MT, TH, AI, HK and TO participated in data collection and the care for the participants. SS and TO performed the statistical analysis. All authors read and approved the final manuscript.

## Supplementary Material

Additional file 1 **Stepwise multiple regression analyses to predict the changes in health status scores**. Tables S1 and S2 showing the stepwise multiple regression analyses used to predict the changes in health status scores. Click here for file
