# Supplementary material for: Effect of exacerbations on health status in subjects with chronic obstructive pulmonary disease
Source: Health Qual Life Outcomes. 2009 Jul 22;7:69. doi: 10.1186/1477-7525-7-69 (PMC2723082; doi:10.1186/1477-7525-7-69)
Supplement: Additional file 1 — Stepwise multiple regression analyses to predict the changes in health status scores. Tables S1 and S2 showing the stepwise multiple regression analyses used to predict the changes in health status scores. [file 1477-7525-7-69-S1.doc]

| **Table S1. Stepwise multiple regression analyses to predict the changes in health status scores^*^** | | | | | | | | | | |
| --- | --- | --- | --- | --- | --- | --- | --- | --- | --- | --- |
|  |  | CRQ | | | |  | SGRQ | | | |
|  |  | Dyspnea | Fatigue | Emotion | Mastery | Total | Symptoms | Activity | Impacts | Total |
| Age |  | - | - | - | - | - | - | - | - | - |
| pre-bronchodilator FEV_1_ |  | - | - | - | - | - | - | - | - | - |
| Current/former-smokers |  | - | - | - | - | - | - | - | - | - |
| RV/TLC |  | - | - | - | - | - | - | - | 0.03 | - |
| KCO |  | - | - | - | - | - | - | - | - | - |
| Baseline scores** |  | 0.04 | 0.06 | 0.04 | 0.11 | - | 0.10 | 0.06 | 0.06 | 0.04 |
| Frequency of exacerbations |  | - | - | - | 0.03 | - | 0.05 | - | - | - |
| Cumulative R^2^ |  | 0.04 | 0.06 | 0.04 | 0.14 | 0.00 | 0.15 | 0.06 | 0.09 | 0.04 |
| * All values listed represent statistically significant coefficients of determination (R^2^) (p<0.05); missing values indicate that the independent variables were not statistically significant. ** Baseline score means the score for the relevant subscale of the CRQ or SGRQ at baseline. | | | | | | | | | | |

| **Table S2. Stepwise multiple regression analyses to predict the changes in health status scores^*^** | | | | | | | | | | |
| --- | --- | --- | --- | --- | --- | --- | --- | --- | --- | --- |
|  |  | CRQ | | | |  | SGRQ | | | |
|  |  | Dyspnea | Fatigue | Emotion | Mastery | Total | Symptoms | Activity | Impacts | Total |
| Age |  | - | - | - | - | - | - | - | - | - |
| pre-bronchodilator FEV_1_ |  | - | - | - | - | - | - | - | - | - |
| Current/former-smokers |  | - | - | - | - | - | - | - | - | - |
| RV/TLC |  | - | - | - | - | - | - | - | 0.03 | - |
| KCO |  | - | - | - | - | - | - | - | - | - |
| Baseline scores** |  | 0.04 | 0.06 | 0.04 | 0.11 | - | 0.10 | 0.06 | 0.06 | 0.04 |
| Frequency of exacerbations |  | - | - | - | 0.03 | - | 0.05 | - | - | - |
| Cumulative R^2^ |  | 0.04 | 0.06 | 0.04 | 0.14 | 0.00 | 0.15 | 0.06 | 0.09 | 0.04 |
| * All values listed represent statistically significant coefficients of determination (R^2^) (p<0.05); missing values indicate that the independent variables were not statistically significant. ** Baseline score means the score for the relevant subscale of the CRQ or SGRQ at baseline. | | | | | | | | | | |
